# What is critical metascience and why is it important?

**DOI:** 10.3389/fpsyg.2026.1860740

**Published:** 2026-07-02

**Authors:** Mark Rubin

**Affiliations:** Department of Psychology, Durham University, Durham, United Kingdom

**Keywords:** metascience, open science, questionable research practices, replication crisis, science reform

## Abstract

Metascience uses a scientific approach to understand and improve science. Critical metascience takes a step back to question metascience’s common assumptions, methods, problems, and solutions. In this article, I aim to raise the profile of critical metascience and offer a rationale for viewing it as a distinct area of multidisciplinary inquiry. I define critical metascience, explain why it is important, and address some potential concerns about it. I also consider some emerging themes in the area and evaluate its potential relationships with metascience. I conclude that there needs to be greater recognition of critical metascience and greater interaction between metascience and critical metascience in order to improve metascience’s objectivity and approach.

## What is metascience?

Modern metascience took off in the 2010s in response to concerns about research integrity and low replication rates, and it has been “exploding” in recent years [Tim Errington, quoted in Metascience Can Improve Science (2025)]. But what is metascience? There are several definitions (UK Metascience Unit, 2025, pp. 4–5). One helpful approach is to locate it at the intersection of the science of science, open science, and methodological activism (Peterson and Panofsky, 2023, p. 159).

It is also useful to distinguish between three types of metascience. *Basic metascience* uses a scientific approach to understand and critique the way we do science. *Applied metascience* designs and tests interventions to improve science. Finally, *activist metascience* focuses on reforming research culture by changing norms, incentives, and policies in line with open science principles (e.g., Grant et al., 2025; Nosek, 2019; Nosek et al., 2012; Peterson and Panofsky, 2021b). Importantly, this “activist reform movement” (Vazire and Nosek, 2023, p. 3) dominates modern metascience, setting the agenda for both basic and applied metascience (Bastian, 2021; Nielsen and Qiu, 2018; Peterson and Panofsky, 2021a, 2023).

## What is critical metascience?

**Figure 1:**
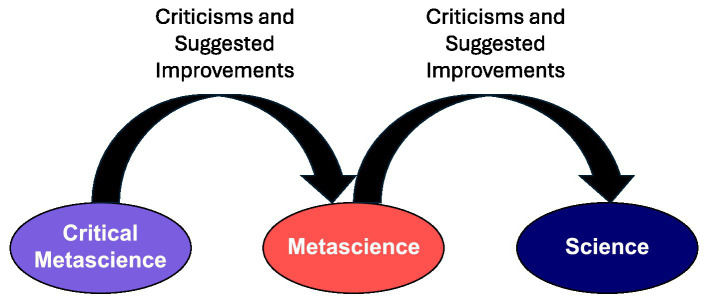
The relationship between critical metascience, metascience, and science.

I define critical metascience as a multidisciplinary research area that takes a step back to question some of metascience’s commonly accepted assumptions, methods, problems, and solutions. For this reason, critical metascience has also been described as “meta-meta-science” (Quintana and Heathers, 2023), and that term works well because it captures the idea that, if science is the subject of metascience, then metascience is the subject of critical metascience. However, the “critical” part of critical metascience means that we are not only *studying* metascience; we are also actively *criticizing* some of its key assumptions and making suggestions for improvements, as shown in Figure 1.

So, “critical metascience” does not mean “metascientists who are critical” because metascientists are critical of themselves and others all the time. Instead, “critical metascience” refers to a special type of criticism that challenges metascience’s dominant ideas and assumptions and offers new, countervailing perspectives. Hence, the word “critical” refers to radical, disruptive, *paradigmatic criticism* (e.g., “Questionable research practices [QRPs] are not necessarily problematic”) rather than internal, *conventional criticism* that implicitly accepts the paradigm’s assumptions (e.g., “This measure of QRPs is unreliable”).

Paradigmatic criticism is important because it can help to highlight conceptual issues that are not obvious from within a paradigm. As Dewey explained, “failure to examine the conceptual structures and frames of reference which are unconsciously implicated in even the seemingly most innocent factual inquiries is the greatest single defect that can be found in any field of inquiry” (Dewey, 1981, p. 501).

Importantly, paradigmatic criticism can lead to constructive suggestions for improvement. To give three examples, (a) qualitative researchers have argued that researcher positionality and reflexivity can be used to address the open science principle of transparency (e.g., Field and Pownall, 2025; Steltenpohl et al., 2023); (b) computational modelers have argued that formalizing theories can facilitate “open theory” in parallel with open data and research materials (Guest and Martin, 2021); and (c) “pre-replication” assessments have been proposed to check whether replication studies are worthwhile given epistemic goals and the methodological quality of the original studies (Karhulahti et al., 2025).

There is plenty of work that fits the definition of critical metascience. For example, Rubin (2026b) has provided a list of 200 + articles in this area. There has also been some formal recognition of “critical metascience” in journal articles and conference symposia (e.g., Lohse, 2026; Ulpts and Bartscherer, 2025). However, critical metascience is not yet widely recognized as constituting an interrelated body of work. In the current article, I aim to raise the profile of this research area and offer a rationale for viewing it as a distinct field of multidisciplinary inquiry.^1^ To do so, I focus on work published from 2012 onwards that discusses science reform initiatives in the context of the replication crisis in psychology and beyond.

## Why is critical metascience important?

Critical metascience is important because it can help to highlight and address collective biases in metascience. Of course, all scientific fields have biases. However, metascience may be particularly prone to collective biases because it is heavily influenced by its activist open science reform movement.

Hauswald (2021) work is informative here. He considered how collective biases might occur among scientists who are affiliated with the social activist movement that they study (e.g., climate activism, feminism, animal rights). As he explained:

“If … the community of an entire research field is dominated by scholars with a particular activist affiliation … [and] if many or all members are biased in the same direction, their biases accumulate, as it were, to a collective bias. From a pluralist point of view, a dominance of persons with a particular activist background appears to be epistemically *detrimental* insofar as it reduces epistemic diversity. Research fields that are intimately connected to particular activist movements therefore seem to be particularly prone to such collective biases” (p. 612, emphasis in original).

Based on Hauswald’s (2021) argument, collective biases may also be present in metascience. Indeed, they may be particularly strong in this area given that activist metascientists are inextricably linked to a science reform movement that originated from within metascience, rather than being coincidentally affiliated with an external social movement. Hence, they are likely to be especially invested in reform efforts and the assumptions that underpin those efforts (e.g., a science-wide replication crisis). This is not to dispute the good intentions of activist metascientists. It is only to point out that the collective dominance of the assumptions behind those intentions may have a detrimental effect on the field’s epistemic diversity and increase its propensity for collective biases [Bashiri et al., 2025, p. 9; Bazzoli, 2022; Rubin, 2023a; Rubin, 2023b, pp. 7–9; see also Teymoori and Trappes (2026, p. 11)].

Hauswald (2021) also predicted “the presence of various social mechanisms that produce or reinforce collective biases in such fields” (p. 612). For example, De Melo-Martín and Intemann (2014) have considered how scientists might engage in *masking*, *discrediting*, and *silencing* when they encounter dissent. Sure enough, some of these strategies have been observed in metascience (e.g., Bastian, 2021; Flis, 2022; Gervais, 2021; Hamlin, 2017, p. 692; Malich and Rehmann-Sutter, 2022, p. 265; Rubin, 2023b; Walkup, 2021; Whitaker and Guest, 2020). For example, in a piece titled “the metascience movement needs to be more self-critical,” Bastian (2021) noted that:

‘Us and them’ thinking and movement camaraderie lead some people to see criticism of fellow-travelers as ‘friendly fire’ that’s out of line, leading them to keep criticisms to themselves. Advocates can also be very quick to jump into defensive attack mode instead of reflection when anyone criticizes. The combination of deterring incisive analysis and not engaging deeply with confronting thoughts is a shortcut to blinding yourself to movement and theory errors.

And this is where I think critical metascience can be helpful, because it deliberately steps back to reconsider some of metascience’s common assumptions, including implicit background assumptions that may be invisible from within the movement’s dominant research culture (Dewey, 1981, p. 501; Kim et al., 2000, p. 68; Longino, 1990, p. 80; Popper, 1983, pp. 17–18; Popper, 1994, p. 135; Teymoori and Trappes, 2026).

To be clear, I am not arguing that critical metascientists are *less* biased than activist metascientists. I am only arguing that they tend to have *different* and *more diverse* biases, and that it may be useful to take their perspectives into account in order to increase epistemic diversity (Hauswald, 2021; Hull, 1988; Longino, 1990; O’Rourke-Friel, 2025; Rubin, 2023a). Similarly, I am not arguing that activist metascience cannot improve science, or that critical metascientists cannot also be activists. I am only arguing that activist metascience is particularly susceptible to collective biases about what “science” is and how to “improve” it, and critical metascience can help to highlight and address these biases.

## Questioning some common metascience assumptions

**Figure 2:**
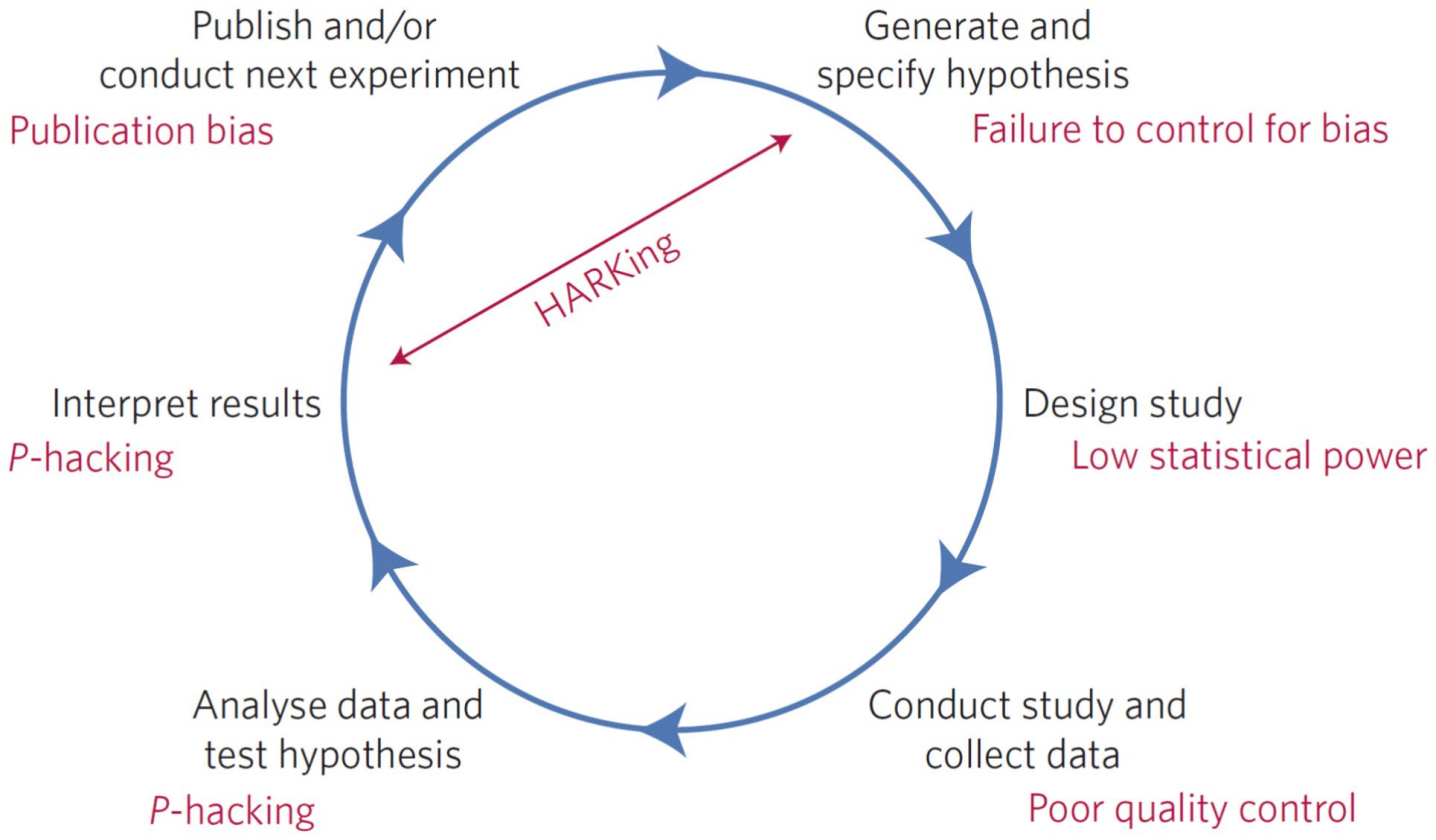
Munafò et al.’s (2017) proposed threats to the hypothetico-deductive method.

To illustrate some of metascience’s common assumptions, consider this famous diagram from Munafò et al.’s (2017) “Manifesto for Reproducible Science” (Figure 2; see also Chambers et al., 2014).

According to Munafò et al.’s (2017), researcher bias, low statistical power, HARKing, *p*-hacking, and publication bias lead to false positives and, consequently, low replication rates. Now, there is certainly good evidence and reasonable arguments to warrant these sorts of concerns. For example, metascientists and others often agree that:

1. Exploratory results are more “tentative.” E.g., Hardwicke and Wagenmakers (2023); Nelson et al. (2018); Nosek and Lakens (2014); Simmons et al. (2021b); Wagenmakers et al. (2012).
2. QRPs are problematic. E.g., Agnoli et al. (2017); Banks et al. (2016); Fraser et al. (2018); Hartgerink and Wicherts (2016); John et al. (2012); Miller et al. (2025); Pickett and Roche (2018).
3. E.g., 1: HARKing is problematic. E.g., Bishop (2019); Bosco et al. (2016); Kerr (1998); O’Boyle et al. (2014).
4. E.g., 2: *p*-hacking is problematic. E.g., Bishop (2019); Nagy et al. (2025); O’Boyle and Götz (2022); Reis and Friese (2022); Simmons et al. (2011); Stefan and Schönbrodt (2023); Wicherts et al. (2016).
5. Publication bias is problematic. E.g., Bishop (2019); Chambers and Tzavella (2022); Errington et al. (2021); Schimmack (2020).
6. Low replication rates are problematic. E.g., Baker (2016); Open Science Collaboration (2015); Munafò et al. (2017); Pashler and Wagenmakers (2012); Schimmack (2020); Wingen et al. (2020).

These six claims are now so widely accepted within metascience that they are treated as almost indisputable facts. In this sense, they form part of an irrefutable Lakatosian “hard core” in the metascience movement’s research program (Lakatos, 1978). Critically, however, Lakatosian research programs grow by qualifying their hard cores with auxiliary hypotheses that accommodate anomalies and make successful new predictions (e.g., “low replication rates are problematic in Situation X but not in Situation Y”; Lakatos, 1978, pp. 21, 50, 179). The problem is that this sort of theoretical progress appears to be lacking, or at least stunted, in metascience, suggesting that the field may be in a pseudoscientific state (Lakatos, 1978, pp. 33–34). In particular, there seems to be a lack of appetite in activist metascience to consider the limits of these six claims.

Metascience’s meagre theoretical progress cannot be attributed to the absence of important empirical and theoretical work that has addressed these issues. For example, there is now good evidence and arguments that challenge the generality of each of the previous six claims and call for some nuance in their articulation [for a review, see Rubin (2026a)]:

1. Exploratory results are more “tentative.” But see Devezer et al. (2021); Feest and Devezer (2025); Rubin (2017a); Rubin and Donkin (2024); Szollosi and Donkin (2021).
2. QRPs are problematic. But see Fiedler and Schwarz (2016); Linder and Farahbakhsh (2020); Moran et al. (2022, Table 6); Ravn and Sørensen (2021); Rubin (2023b); Sacco et al. (2019); Ulrich and Miller (2020).
3. E.g., 1: HARKing is problematic. But see Leung (2011); Oberauer and Lewandowsky (2019); Mohseni (2020); Rubin (2017b, 2022); Vancouver (2018).
4. E.g., 2: p-hacking is problematic. But see Adda et al. (2020); Erasmus (2024); Gupta and Bosco (2023); Hartgerink (2017); Reinagel (2023); Rooprai et al. (2023); Rubin (2024, 2026c); Stanley et al. (2018); Ulrich and Miller (2020); Wegener et al. (2024).
5. Publication bias is problematic. But see Brodeur et al. (2023); Dalton et al. (2012); Frankel and Kasy (2022); Linden et al. (2024); Mathur and VanderWeele (2021); Ramos (2025); Van Aert et al. (2019).
6. Low replication rates are problematic. But see De Boeck and Jeon (2018); Devezer et al. (2019, 2021); Devezer and Buzbas (2026); Fanelli (2018); Feest (2019); Firestein (2012, 2016); Greenfield (2017); Guttinger (2020); Haig (2022); Iso-Ahola (2020); Leonelli (2018); Lewandowsky and Oberauer (2020); Pethick et al. (2025); Rubin (2023b, 2025a); Stroebe and Strack (2014).

Take *p*-hacking as an example. There is now work showing that it is not as prevalent as commonly assumed (Adda et al., 2020, p. 29; Fiedler and Schwarz, 2016; Gupta and Bosco, 2023; Hartgerink, 2017; Rooprai et al., 2023; Stanley et al., 2018); that it only inflates Type I error rates in some philosophies of significance testing (Rubin, 2024, 2026c); and that it may even be beneficial by increasing researchers’ chances of detecting true positives (Erasmus, 2024; Reinagel, 2023; Ulrich and Miller, 2020).

Isn’t this type of work just standard self-critical metascience in action? Perhaps it should be, and maybe some of it is! However, if it was genuinely accepted as standard metascience, then it would be met with less defensiveness (Bastian, 2021; Flis, 2022; Gervais, 2021; Hamlin, 2017; Malich and Rehmann-Sutter, 2022, p. 265; Walkup, 2021), and its conclusions would be better integrated into the field to produce more cautious, complex, and nuanced models of science (Greenwald et al., 1986, pp. 223–224; Lakatos, 1978, p. 50; Russell, 1912, p. 8). Instead, with only a few exceptions, “critical” work like this tends to be less acknowledged than standard metascience (Flis, 2022; Horbach et al., 2024; Rubin, 2023b), and relatively simplistic models endure (Flis, 2019, p. 170; Peterson and Panofsky, 2023, p. 169; Pham and Oh, 2021b, p. 168). This is a shame because it is precisely this type of work that we should be paying attention to if we want to tailor the right kinds of science reforms to the right areas and implement them in the right ways.

To illustrate the problem, consider John et al.’s (2012) widely cited study on questionable research practices. They found that “the percentage of respondents who have engaged in questionable practices was surprisingly high” (p. 524). In response, Fiedler and Schwarz (2016) conducted a follow-up study and concluded that John et al.’s (2012) work “suffers from ambiguities that prohibit the damning conclusions drawn” (p. 50). More recently, Horbach et al. (2024) compared 450 citation contexts in which these two studies were cited and found that:

“The total number of citation contexts showing awareness of Fiedler and Schwarz’ criticisms (18/450) is extremely low …. Similarly, the number of citation contexts mentioning other critiques (5/450) or in any way critically engaging with the content of the John et al. (2012) study (29/450) is remarkably low” (p. 14).

Horbach et al. (2024) concluded that there is “an apparent lack of critical engagement with the cited literature, leading to incorrect reproductions of claims and overgeneralisation of supposed research findings” (p. 1). Commenting on a similar example involving an article that was critical of preregistration, Rubin (2023b) noted that “this type of citation bias creates an illusion of consensus in the literature, and it may obstruct the motive for theory improvement by giving the impression that current theories are adequate and undisputed” (p. 2).

In practice then, “critical” work that questions common metascience assumptions receives relatively low engagement within metascience. This may be because its calls for caution, caveats, and nuance are viewed as challenging activist metascience’s more universalist claims of a science-wide “crisis” and the need for science-wide reforms (Jamieson, 2018; Penders, 2024, p. 3). Again, it is important to stress that critical metascience is not necessarily anti-science reform in this respect. It may be in favour of reforms that have greater contextualization, differentiated implementation, and/or acknowledgment of domain-specific standards.

## Why does metascience need critical metascience?

But why does metascience need a special “critical” research field? What makes it different from other disciplines in this respect? Well, in fact, some other disciplines also have critical fields. For example, psychology has *critical psychology* (Parker, 2007) and, more recently, the field of *critical data studies* has arisen to question dominant views in data science (Iliadis and Russo, 2016). Notably, both of these fields adopt a critical theory approach that examines power structures, ideologies, and social injustices within their respective disciplines (Bohman, 2021). Although some critical metascience may follow a similar approach, I view the area as being broader than, and not constrained to, critical theory. Hence, although critical metascience may consider how metascience reinforces certain power structures, ideologies, and social injustices, it may also address purely philosophical, statistical, or methodological issues in metascience.

There are also some unique aspects of metascience that make critical metascience particularly germane. First, modern metascience arose in response to a “crisis” about replication and research integrity (Nielsen and Qiu, 2018; Pashler and Harris, 2012; Schooler, 2014). The problem is that, in a crisis, people often respond with a biased cognitive style. For example, they may experience a range of negative moral emotions, have a narrow focus on specific issues, submit to autocratic decision-making, engage in consensus-seeking and groupthink, and feel a pressure to implement solutions urgently (e.g., Antonetti et al., 2025; Paulus et al., 2022; Turner and Pratkanis, 1998). Consequently, it is useful to solicit the views of people who are not operating in this crisis mode.

Second, metascience is heavily influenced by psychologists because psychology was ground zero for the replication crisis (Open Science Collaboration, 2015; for a review, see Teymoori and Trappes, 2026). This influence may give the field a somewhat unrepresentative view of science and its problems (Flis, 2019; Malich and Rehmann-Sutter, 2022; Moody et al., 2022; Morawski, 2022). Consequently, as Gervais (2021) argued, “a movement that has emerged from critical reflection on psychological science should be open to critical self-reflection on its own workings and open to wisdom and critiques from other fields that may have important theoretical insights” (p. 828; see also Hamlin, 2017, p. 691). Critical metascience’s multidisciplinary approach may facilitate more radical, paradigmatic criticism from other fields that helps to provide these valuable insights.

Finally, activist metascience can be construed as a moral crusade to save science from self-interested scientists who are operating in a dysfunctional incentive system (Bartscherer, 2026; Bastian, 2021; Knibbe et al., 2025; Morawski, 2020; Nosek et al., 2012; Penders, 2024; Peterson and Panofsky, 2023; Robinson-Greene, 2017). According to this view, activist metascientists may sometimes experience a “white hat bias” (Lilienfeld, 2020, p. 2) that leads them to feel morally justified in summarily ignoring or dismissing criticisms and rebuttals from what they perceive to be an “old guard” of scientists (e.g., prominent psychology professors) who are invested in protecting their powerful positions within the status quo (Derksen and Field, 2022, p. 172; Flis, 2022, p. 147). By bringing in less invested experts from other disciplines (e.g., statistics, the philosophy of science, and science and technology studies), critical metascience can help to add legitimacy to valid critical arguments.

## Some counterarguments considered

It is useful to consider some counterarguments to the proposal for a distinct research area of “critical metascience.” First, if metascience benefits from critical metascience, then surely critical metascience should benefit from its own paradigmatic criticism because, as it develops, it is likely to form certain theoretical and ideological commitments, methodological preferences, academic incentives, group dynamics, and collective biases that can only be recognized from an external perspective. For example, critical metascience may develop a tendency toward contrarianism, skepticism of reform, and/or suspicion of methodological standardization. Consequently, we may need a “meta-meta-meta-science” to adequately criticize critical metascience. Reiterating this argument at each meta-level, we would then end up with an infinite progression of metafields.

I agree that, like other fields, critical metascience will benefit from its own external critique (for some initial criticisms, see Syed, 2025). Indeed, if it develops into a more integrated field, then it may even warrant its own metafield of “meta-meta-meta-science.” However, there is likely to be a law of diminishing returns as we progress from one metafield to the next, because each new metafield will criticize a subject that is increasingly distant from the primary target of science. For example, criticizing the field that criticizes the field that criticizes metascience will have only very indirect implications for science. This law of diminishing returns may help to explain why the progression of metafields tends to reach a natural limit in other areas. For example, although there are disciplines of history, historiography, and the philosophy of historiography, there is no “philosophy of the philosophy of historiography” (Tucker, 2004).^2^

Second, metascience is a relatively heterogeneous field that encompasses different disciplines, philosophies, approaches, and agendas (Field, 2022; Uygun Tunç et al., 2023). Critical metascience’s paradigmatic criticism runs the risk of artificially homogenizing this diversity (Rubin, 2023b; Syed, 2025). Notably, a similar concern has been raised with regard to metascience’s critique of contemporary science (Guttinger, 2020, p. 2; Malich and Rehmann-Sutter, 2022; Peterson and Panofsky, 2023, p. 166). For example, Malich and Rehmann-Sutter (2022) argued that “metascience often construes science and psychology from a homogenizing perspective that omits the plurality and complexity of both scientific methods and psychological approaches” (p. 269). In both cases, the solution is not to throw up our hands and abandon paradigmatic criticism entirely but, instead, to engage in a more careful, nuanced, and constrained form of this criticism that clearly acknowledges its limitations. For example, in the present article, I have focused on activist metascience and the impact of its open science reform movement on basic and applied metascience. However, I readily concede that not all basic and applied metascience is influenced by activist metascience to the same extent. At the end of the day, if metascience is able to accomplish a nuanced critique of the sprawling enterprise of contemporary science, then critical metascience should be able to undertake a nuanced critique of the smaller and more focused area of contemporary metascience.

Third, even if metascience benefits from external criticism, do we really need a new research field to do the job? Are not established disciplines such as the philosophy of science and science and technology studies (STS) sufficient? Again, this question of potential redundancy has also been raised with respect to metascience: Critics have argued that a separate field of “metascience” is unnecessary because science is already sufficiently self-critical and self-correcting. As one reviewer of this article argued, “if science can’t police itself, it isn’t science.” In response, metascientists have argued that it remains useful to develop a dedicated field of scientific research that treats science as an object of study in order to identify and address broader systemic issues that impact its “efficiency” [Nosek et al., 2012, p. 619; see also Flis (2022), Peterson and Panofsky (2021b, p. 584), and Vazire and Holcombe (2022)]. The rationale for critical metascience is similar; it is just pitched at a higher level of abstraction. Hence, it is accepted that metascience includes a degree of internal, conventional self-criticism. However, it is argued that critical metascience can make an important contribution by treating metascience as an object of study in order to identify and address systemic, paradigmatic issues that may not be as visible from inside the field. From this perspective, it would be inconsistent to argue that we need a distinct research area to study, criticize, and improve the way we do science (i.e., metascience), but that we do not need a distinct research area to study, criticize, and improve the way we do metascience (i.e., critical metascience). Furthermore, like metascience, critical metascience does not aim to replace established disciplines such as the philosophy of science and STS. Instead, it is intended to be way of organizing and integrating these and other disciplines into a more focused and interdisciplinary approach to the study of metascience.

Finally, it is possible that critical metascience arguments may be misappropriated by bad actors who want to sabotage science reform in order to further their anti-science agenda. I agree that this is a danger. However, this point should not be used as a justification for ignoring critical views. If we go down that road, then, to be consistent, we should also ignore activist metascientists’ criticisms of the status quo, because their narrative about the replication “crisis” and the need for urgent science-wide “reform” can also be co-opted by bad actors for anti-science reasons (Biagioli and Pottage, 2022; Jamieson, 2018; Levy and Johns, 2016; Nosek, 2025; O’Grady, 2025). Hence, both critical and activist metascience arguments run the risk of misappropriation, but that danger should not lead us to suppress or ignore those arguments. It should only caution us to be *careful* about how we communicate our arguments in order to reduce the potential for their misuse.

## Some emerging themes in critical metascience

Unlike metascience, critical metascience does not yet have a coherent narrative (e.g., QRPs cause false positives, which led to the replication crisis). Nonetheless, we can identify some emerging themes in the area.

One line of work has questioned the meaning and interpretation of replication failures, arguing that low replication rates (a) may not represent a “crisis” and (b) may not occur throughout science (e.g., Devezer and Buzbas, 2026; Fanelli, 2018; Firestein, 2016; Haig, 2022; Lewandowsky and Oberauer, 2020; Pethick et al., 2025; Stroebe and Strack, 2014).

Another line of work has argued that metascience is too heavily focused on replication, statistics, and methodology, and that this preoccupation may encourage *naïve empiricism*, *statisticism*, and *methodologism, respectively,* (e.g., Haig, 2022; Proulx and Morey, 2021; Rubin, 2023b; van Rooij and Baggio, 2021). This sort of work often calls for a greater focus on theory and theory development (e.g., Dames et al., 2024; Guest and Martin, 2021; Lavelle, 2023; Szollosi and Donkin, 2021).

There is also work questioning the rationale for and implementation of various open science reforms, such as replication, preregistration, and open data (e.g., Berberi and Roche, 2022; Haig, 2022; Hicks, 2023; Navarro, 2020; Prosser et al., 2023; Rubin, 2020). One concern here is that these research practices may not translate well across diverse scientific disciplines and paradigms (e.g., Khan et al., 2024; Lamb et al., 2024; Lohse, 2026; Lash and Vandenbroucke, 2012; MacEachern and Van Zandt, 2019; Pownall, 2024; Ulpts et al., 2025).

There has also been a continuous stream of articles arguing that metascience has a biased view of science that tends to exclude minority group scientists and approaches (e.g., Bazzoli, 2022; Hamlin, 2017; Hostler, 2024; Leonelli, 2022; Ulpts, 2024; Whitaker and Guest, 2020). Finally, there is an important line of work that examines the sociology of the metascience movement, including its roots in psychology and its scandalizing and moralizing tendencies (e.g., Flis, 2019; Penders, 2024; Peterson and Panofsky, 2021a, 2023).

There are further examples of these and other themes in Rubin’s (2026b) list of 200 + critical metascience articles. Attesting to critical metascience’s multidisciplinary constitution, these articles are authored by statisticians, natural scientists, psychologists, political scientists, sociologists, ethnographers, STS scholars, philosophers and historians of science, and, of course, metascientists. It is likely that many of these scholars do not identify as “critical metascientists” (for a similar issue in the philosophy of historiography, see Tucker, 2004, p. 1). What unites the contributions in this area is not a shared identity but a shared focus on questioning metascience’s commonly accepted assumptions, approaches, problems, and solutions. Hence, just as you do not have to identify as a “metascientist” to do metascience (James Wilsdon, quoted in Stafford, 2025), you do not have to identify as a “critical metascientist” to do critical metascience!

## The relationship between metascience and critical metascience

It is useful to consider the relationships that might exist between metascience and critical metascience because they inform the potential for fruitful cooperation between these two research areas. Here, I consider four possibilities.

**Figure 3:**
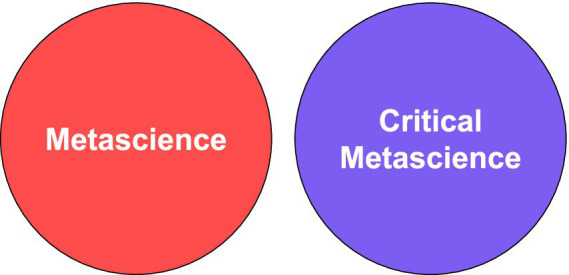
Metascience and critical metascience as two separate fields.

First, the two research areas could be considered as being completely separate from one another, as shown in Figure 3.

**Figure 4:**
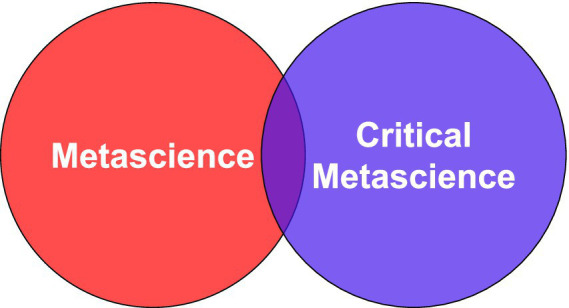
Metascience and critical metascience as partially overlapping fields.

One advantage of this first model is that it is likely to facilitate paradigmatic criticism. As Miller (2025) explained, “only an outsider, who adheres to a different conceptual framework, and isn’t committed to the insider’s standards, can thoroughly criticize the insider’s framework” [p. 44; see also Longino (1990, p. 80)]. The problem with this model is that it may result in quite strong “us” and “them” relations that cause group polarization and intergroup bias (Bastian, 2021; see also Teymoori and Trappes, 2026, p. 11). In addition, the model fails to acknowledge that metascientists may sometimes engage in paradigmatic criticism of metascience themselves (Syed, 2025). Hence, instead, it is useful to consider a model in which there is some limited overlap between the two research areas, as shown in Figure 4.

In this second model, “metascientists” may sometimes step back to criticize some of their field’s core assumptions, and “critical metascientists” may accept some of these core assumptions while criticizing other aspects of metascience. This model continues to facilitate paradigmatic criticism, but it is less exclusionary and separationist than the first model, and it may lead to more productive interactions.

**Figure 5:**
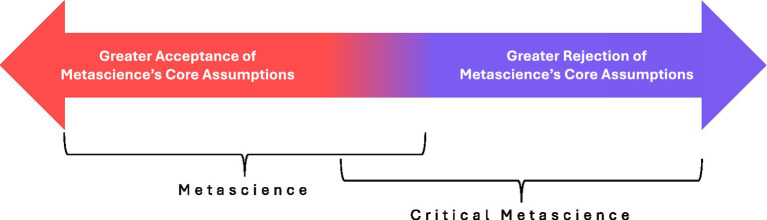
A critical metascience continuum.

Another way to conceive this second model is as a continuum ranging from greater acceptance of metascience’s core assumptions at one end to greater rejection of those assumptions at the other end, as shown in Figure 5.

In this case, more conventional, internal criticism would occur towards the left end of the continuum, and more paradigmatic, external criticism would occur towards the right end. Again, this model is more flexible than the first because it allows the same people to occupy different points along the continuum at different times and for different research projects.^3^

**Figure 6:**
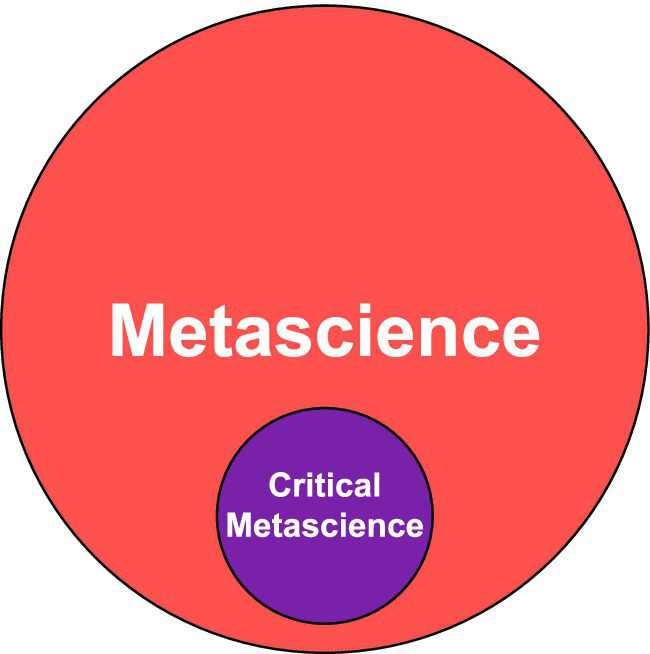
Critical metascience as a subfield of metascience.

Finally, it is worth considering two further models. First, we could view critical metascience as a subfield of metascience, as shown in Figure 6.

**Figure 7:**
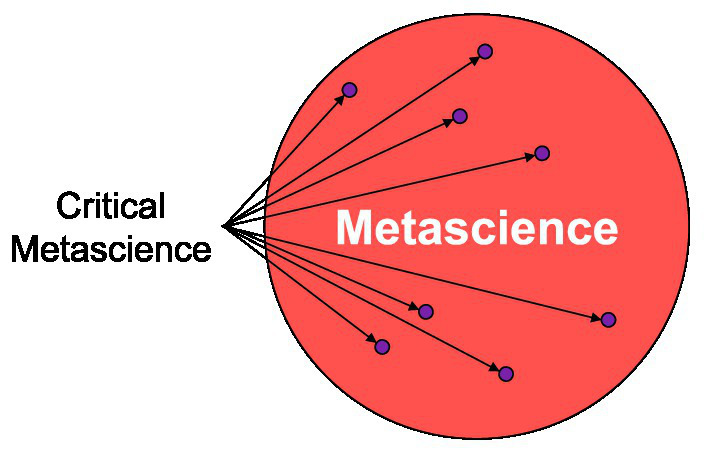
Critical metascience integrated throughout metascience.

Second, we could go further and consider critical metascience as being peppered into all aspects of metascience, as shown in Figure 7.

There are two problems with these last two models. First, logically, it does not make sense to argue that critical metascience is a subfield of, or dispersed throughout, metascience because the two fields have different objects of study. Critical metascience aims to criticize and improve contemporary *metascience*, whereas metascience aims to criticize and improve contemporary *science* (see Figure 1). Hence, if a metascientist decides to engage in radical, disruptive criticism of metascience, rather than science, then, ipso facto, they are doing critical metascience.

Second, the criticism comes from *within metascience* in Figures 6 and 7. Hence, it is more likely to be grounded in metascience’s core assumptions and less likely to challenge those assumptions. Consequently, these last two models are less likely to generate the more radical, paradigmatic criticism that characterizes critical metascience. Indeed, it would be self-contradictory and epistemically incoherent for the majority of self-described “metascientists” to routinely reject their own field’s foundational assumptions.

## What needs to change and why?

To conclude, I would like to address the questions that were posed by the convenors of the symposium on “Critical Metascience” that inspired this article (Ulpts and Bartscherer, 2025): “What needs to change and why?” In my view, there should be greater recognition of critical metascience and greater interaction between metascience and critical metascience in order to improve metascience’s objectivity and approach.

For its part, metascience champions scientific criticism. For example, it advocates adversarial collaborations, red teams, effective peer review, error detection, and self-correction, and it encourages scientists to “bend over backwards to show how [they]’re maybe wrong” (Feynman, 1974). Consequently, it should welcome critical metascience as a useful addition to its own self-criticism toolkit. Of course, metascientists do not always need to concede to critical metascience arguments, but they should at least engage with those arguments publicly, formally, and carefully (Longino, 1990, p. 78; O’Rourke-Friel, 2025; Rubin, 2023b).

In turn, critical metascientists should be prepared to reach out to metascientists in order to have productive exchanges rather than to criticize from afar. As discussed previously, they should also be careful not to artificially homogenize metascience or criticize straw-person or outdated versions of metascience arguments (Field, 2022; Rubin, 2023b; Syed, 2025; Uygun Tunç et al., 2023). Finally, they should be reflexive, continuously questioning their own perspectives, biases, and positionality (Bashiri et al., 2025, p. 7).

It is worth noting that productive dialogue between metascientists and critical metascientists may not always be easy. In particular, some critical metascience arguments may be unintentionally distorted and misrepresented when they are interpreted through the lens of metascience’s dominant paradigm. Popper (1983) noted a similar problem in the area of philosophy:

“Thinking people tend to develop some framework into which they try to fit whatever new idea they may come across; as a rule, they even translate any new idea which they meet into a language appropriate to their own framework. One of the most characteristic tasks of philosophy is to attack, if necessary, the framework itself. And in order to do so, it may become necessary to attack beliefs which, whether or not they are consciously held, are taken so much for granted that any criticism of them is felt to be perverse or insincere. Whenever the framework itself is attacked, its defenders will as a rule interpret, and attempt to refute, the attack within their own adopted framework. But in trying to translate critical arguments directed against the framework into a language appropriate to that framework, they are liable to produce fatal distortions and misunderstandings” (pp. 17–18).

The solution here is clearer communication and interpretation on both sides. As Popper (2002) explained, “we should always try to clarify and to strengthen our opponent’s position as much as possible before criticizing [them], if we wish our criticism to be worth while” (p. 258).

Following this respectful approach, productive interactions between metascientists and critical metascientists might take the form of research collaborations, discussions, and debates in journals, blogs, and conferences. A good example can be found in the journal-based debate on the merits of preregistration that took place between Pham and Oh (2021a, 2021b) and Simmons et al. (2021a, 2021b).

Why is this increased dialogue useful? It can help to improve metascience by making it more objective. Again, Popper’s (1976) view is relevant:

“Scientific objectivity is based solely upon a critical tradition which, despite resistance, often makes it possible to criticize a dominant dogma. To put it another way, the objectivity of science is … [the result] of the friendly-hostile division of labour among scientists” (p. 95).

Critical metascience can play an important part in Popper’s “friendly-hostile division of labour” by offering a special type of external criticism that exposes and challenges potential biases and dogma in metascience in order to improve its objectivity and approach.

## Footnotes

1 Throughout this article, I use the terms “research area” and “research field” interchangeably. Other less common but perhaps more accurate terms are also available. For example, the UK Metascience Unit (2025) has considered conceptualizations of metascience as a “discourse coalition” and a “source of collective intelligence” (p. 31). Each of these terms may also be used to describe critical metascience.

2 I am grateful to an anonymous reviewer for suggesting this point.

3 Consistent with Figure 5’s continuum model, some of the articles in Rubin’s (2026b) list of critical metascience articles are less radical and disruptive than others.

## References

[ref1] Adda J. Decker C. Ottaviani M. (2020). P-hacking in clinical trials and how incentives shape the distribution of results across phases. Proc. Natl. Acad. Sci. 117, 13386–13392. doi: 10.1073/pnas.1919906117, 32487730 PMC7306753

[ref2] Agnoli F. Wicherts J. M. Veldkamp C. L. Albiero P. Cubelli R. (2017). Questionable research practices among Italian research psychologists. PLoS One 12:e0172792. doi: 10.1371/journal.pone.0172792, 28296929 PMC5351839

[ref3] Antonetti P. Valor C. Božič B. (2025). Mitigating moral emotions after crises: a reconceptualization of organizational responses. J. Bus. Ethics 203, 787–805. doi: 10.1007/s10551-025-06042-5

[ref4] Baker M. (2016). 1,500 scientists lift the lid on reproducibility. Nature 533, 452–454. doi: 10.1038/533452a, 27225100

[ref5] Banks G. C. Rogelberg S. G. Woznyj H. M. Landis R. S. Rupp D. E. (2016). Evidence on questionable research practices: the good, the bad, and the ugly. J. Bus. Psychol. 31, 323–338. doi: 10.1007/s10869-016-9456-7

[ref6] Bartscherer S. F. (2026). Cultures of trial and error: the narrative side of (open) science. Cultures of Trial and Error. doi: 10.36850/448e9a73-75b2

[ref7] Bashiri F. Perez Vico E. Hylmö A. (2025). Scholar-activism as an object of study in a diverse literature: preconditions, forms, and implications. Humanit. Soc. Sci. Commun. 12, 1–14. doi: 10.1057/s41599-025-05573-6

[ref8] Bastian H. (2021). The metascience movement needs to be more self-critical. PLOS blogs: absolutely maybe. Available online at: https://absolutelymaybe.plos.org/2021/10/31/the-metascience-movement-needs-to-be-more-self-critical/

[ref9] Bazzoli A. (2022). Open science and epistemic pluralism: a tale of many perils and some opportunities. Ind. Organ. Psychol. 15, 525–528. doi: 10.1017/iop.2022.67

[ref10] Berberi I. Roche D. G. (2022). No evidence that mandatory open data policies increase error correction. Nature Ecology Evolution 6, 1630–1633. doi: 10.1038/s41559-022-01879-9, 36109655

[ref11] Biagioli M. Pottage A. (2022). Dark transparency: hyper-ethics at trump’s EPA. Los Angeles Review of Books 38. Available online at: https://lareviewofbooks.org/article/dark-transparency-hyper-ethics-at-trumps-epa/

[ref12] Bishop D. V. (2019). Rein in the four horsemen of irreproducibility. Nature 568, 435–436. doi: 10.1038/d41586-019-01307-2, 31019328

[ref13] Bohman J. (2021). “Critical theory” in The Stanford Encyclopedia of Philosophy. eds. Zalta E. N. Nodelman U.. Available online at: https://plato.stanford.edu/archives/spr2021/entries/critical-theory/

[ref14] Bosco F. A. Aguinis H. Field J. G. Pierce C. A. Dalton D. R. (2016). Harking's threat to organizational research: evidence from primary and meta-analytic sources. Pers. Psychol. 69, 709–750. doi: 10.1111/peps.12111

[ref15] Brodeur A. Carrell S. Figlio D. Lusher L. (2023). Unpacking p-hacking and publication bias. Am. Econ. Rev. 113, 2974–3002. doi: 10.1257/aer.20210795

[ref16] Chambers C. D. Feredoes E. Muthukumaraswamy S. D. Etchells P. (2014). Instead of “playing the game” it is time to change the rules: registered reports at AIMS neuroscience and beyond. AIMS Neurosci. 1, 4–17. doi: 10.3934/Neuroscience.2014.1.4

[ref17] Chambers C. D. Tzavella L. (2022). The past, present and future of registered reports. Nat. Hum. Behav. 6, 29–42. doi: 10.1038/s41562-021-01193-7, 34782730

[ref18] Dalton D. R. Aguinis H. Dalton C. M. Bosco F. A. Pierce C. A. (2012). Revisiting the file drawer problem in meta-analysis: an assessment of published and nonpublished correlation matrices. Pers. Psychol. 65, 221–249. doi: 10.1111/j.1744-6570.2012.01243.x

[ref19] Dames H. Musfeld P. Popov V. Oberauer K. Frischkorn G. T. (2024). Responsible research assessment should prioritize theory development and testing over ticking open science boxes. Meta-Psychology 8. doi: 10.15626/MP.2023.3735

[ref20] De Boeck P. Jeon M. (2018). Perceived crisis and reforms: issues, explanations, and remedies. Psychol. Bull. 144, 757–777. doi: 10.1037/bul0000154, 29771554

[ref21] De Melo-Martín I. Intemann K. (2014). Who’s afraid of dissent? Addressing concerns about undermining scientific consensus in public policy developments. Perspect. Sci. 22, 593–615. doi: 10.1162/POSC_a_00151

[ref22] Derksen M. Field S. (2022). The tone debate: knowledge, self, and social order. Rev. Gen. Psychol. 26, 172–183. doi: 10.1177/10892680211015636

[ref23] Devezer B. Buzbas E. O. (2026). The difference between "replicable" and "not replicable" is not itself scientifically replicable. ArXiv. doi: 10.48550/arXiv.2604.26268

[ref24] Devezer B. Nardin L. G. Baumgaertner B. Buzbas E. O. (2019). Scientific discovery in a model-centric framework: reproducibility, innovation, and epistemic diversity. PLoS One 14:e0216125. doi: 10.1371/journal.pone.0216125, 31091251 PMC6519896

[ref25] Devezer B. Navarro D. J. Vandekerckhove J. Ozge Buzbas E. (2021). The case for formal methodology in scientific reform. R. Soc. Open Sci. 8:200805. doi: 10.1098/rsos.200805, 34035933 PMC8101540

[ref26] Dewey J. (1981) in The later Works: 1925–1953, ed. Boydston J. A., vol. 3 (SIU Press).

[ref27] Erasmus A. (2024). P-hacking: its costs and when it is warranted. Erkenntnis 90, 2929–2950. doi: 10.1007/s10670-024-00834-3, 30311153

[ref28] Errington T. M. Denis A. Perfito N. Iorns E. Nosek B. A. (2021). Reproducibility in cancer biology: challenges for assessing replicability in preclinical cancer biology. eLife 10:e67995. doi: 10.7554/eLife.67995, 34874008 PMC8651289

[ref29] Fanelli D. (2018). Opinion: is science really facing a reproducibility crisis, and do we need it to? Proc. Natl. Acad. Sci. 115, 2628–2631. doi: 10.1073/pnas.1708272114, 29531051 PMC5856498

[ref30] Feest U. (2019). Why replication is overrated. Philos. Sci. 86, 895–905. doi: 10.1086/705451

[ref31] Feest U. Devezer B. (2025). Toward a more accurate notion of exploratory research (and why it matters). PhilSci Arch.

[ref32] Feynman R. P. (1974). Cargo cult science: some remarks on science, pseudoscience, and learning how to not fool yourself. Caltech’s 1974 commencement address. Available online at: https://calteches.library.caltech.edu/51/2/CargoCult.htm

[ref33] Fiedler K. Schwarz N. (2016). Questionable research practices revisited. Soc. Psychol. Personal. Sci. 7, 45–52. doi: 10.1177/1948550615612150

[ref34] Field S. M. (2022). Charting the constellation of science reform. doi: 10.31219/osf.io/udfw4

[ref35] Field S. M. Pownall M. (2025). Subjectivity is a feature, not a flaw: a call to unsilence the human element in science. OSF Preprints. doi: 10.31219/osf.io/ga5fb_v1

[ref36] Firestein S. (2012). Ignorance: How it Drives Science. Oxford University Press.

[ref37] Firestein S. (2016). Why Failure to replicate Findings can Actually be good for Science. LA Times. Available online at: https://www.latimes.com/opinion/op-ed/la-oe-0214-firestein-science-replication-failure-20160214-story.html

[ref38] Flis I. (2019). Psychologists psychologizing scientific psychology: an epistemological reading of the replication crisis. Theory Psychol. 29, 158–181. doi: 10.1177/0959354319835322

[ref39] Flis I. (2022). The function of literature in psychological science. Rev. Gen. Psychol. 26, 146–156. doi: 10.1177/10892680211066466

[ref40] Frankel A. Kasy M. (2022). Which findings should be published? Am. Econ. J. Microecon. 14, 1–38. doi: 10.1257/mic.20190133

[ref41] Fraser H. Parker T. Nakagawa S. Barnett A. Fidler F. (2018). Questionable research practices in ecology and evolution. PLoS One 13:e0200303. doi: 10.1371/journal.pone.0200303, 30011289 PMC6047784

[ref42] Gervais W. M. (2021). Practical methodological reform needs good theory. Perspect. Psychol. Sci. 16, 827–843. doi: 10.1177/1745691620977471, 33513312 PMC8273842

[ref43] Grant S. Corker K. S. Mellor D. T. Stewart S. L. K. Cashin A. G. Lagisz M. . (2025). TOP 2025: an update to the transparency and openness promotion guidelines. MetaArXiv. doi: 10.31222/osf.io/nmfs6_v2 PMC13439764 42557573

[ref44] Greenfield P. M. (2017). Cultural change over time: why replicability should not be the gold standard in psychological science. Perspect. Psychol. Sci. 12, 762–771. doi: 10.1177/1745691617707314, 28972841

[ref45] Greenwald A. G. Pratkanis A. R. Leippe M. R. Baumgardner M. H. (1986). Under what conditions does theory obstruct research progress? Psychol. Rev. 93, 216–229. doi: 10.1037/0033-295X.93.2.216, 3714929

[ref46] Guest O. Martin A. E. (2021). How computational modeling can force theory building in psychological science. Perspect. Psychol. Sci. 16, 789–802. doi: 10.1177/1745691620970585, 33482070

[ref47] Gupta A. Bosco F. (2023). Tempest in a teacup: an analysis of p-hacking in organizational research. PLoS One 18:e0281938. doi: 10.1371/journal.pone.0281938, 36827325 PMC9955613

[ref48] Guttinger S. (2020). The limits of replicability. Eur. J. Philos. Sci. 10, 1–17. doi: 10.1007/s13194-019-0269-1, 30311153

[ref49] Haig B. D. (2022). Understanding replication in a way that is true to science. Rev. Gen. Psychol. 26, 224–240. doi: 10.1177/10892680211046514

[ref50] Hamlin J. K. (2017). Is psychology moving in the right direction? An analysis of the evidentiary value movement. Perspect. Psychol. Sci. 12, 690–693. doi: 10.1177/1745691616689062, 28679082

[ref51] Hardwicke T. E. Wagenmakers E. (2023). Reducing bias, increasing transparency, and calibrating confidence with preregistration. Nat. Hum. Behav. 7, 15–26. doi: 10.1038/s41562-022-01497-2, 36707644

[ref52] Hartgerink C. H. J. (2017). Reanalyzing Head et al. (2015): Investigating the robustness of widespread p-hacking. PeerJ, 5:e3068. doi: 10.7717/peerj.3068, 28265523 PMC5337083

[ref53] Hartgerink C. H. J. Wicherts J. M. (2016). Research practices and assessment of research misconduct. ScienceOpen Research. doi: 10.14293/S2199-1006.1.SOR-SOCSCI.ARYSBI.v1

[ref54] Hauswald R. (2021). The epistemic effects of close entanglements between research fields and activist movements. Synthese 198, 597–614. doi: 10.1007/s11229-018-02047-y

[ref55] Hicks D. J. (2023). Open science, the replication crisis, and environmental public health. Account. Res. 30, 34–62. doi: 10.1080/08989621.2021.1962713, 34330172

[ref56] Horbach S. P. J. M. Aagaard K. Schneider J. W. (2024). Meta-research: how problematic citing practices distort science. MetaArXiv. doi: 10.31222/osf.io/aqyhg

[ref57] Hostler T. J. (2024). Research assessment using a narrow definition of “research quality” is an act of gatekeeping: a comment on Gärtner et al. (2022). Meta-Psychology 8. doi: 10.15626/MP.2023.3764

[ref58] Hull D. L. (1988). Science as a process: An Evolutionary Account of the social and Conceptual Development of Science. University of Chicago Press.

[ref59] Iliadis A. Russo F. (2016). Critical data studies: an introduction. Big Data Soc. 3. doi: 10.1177/2053951716674238

[ref60] Iso-Ahola S. E. (2020). Replication and the establishment of scientific truth. Front. Psychol. 11:2183. doi: 10.3389/fpsyg.2020.02183, 33041887 PMC7525033

[ref61] Jamieson K. H. (2018). Crisis or self-correction: rethinking media narratives about the well-being of science. PNAS 115, 2620–2627. doi: 10.1073/pnas.1708276114, 29531076 PMC5856501

[ref62] John L. K. Loewenstein G. Prelec D. (2012). Measuring the prevalence of questionable research practices with incentives for truth telling. Psychol. Sci. 23, 524–532. doi: 10.1177/0956797611430953, 22508865

[ref63] Karhulahti V. Martončik M. Adamkovic M. (2025). Pre-replication: anything goes, once. Meta-Psychology 9. doi: 10.15626/MP.2024.4155

[ref64] Kerr N. L. (1998). HARKing: hypothesizing after the results are known. Personal. Soc. Psychol. Rev. 2, 196–217. doi: 10.1207/s15327957pspr0203_4 15647155

[ref65] Khan S. Hirsch J. S. Zubida O. Z. (2024). A dataset without a code book: ethnography and open science. Front. Sociol. 9:1308029. doi: 10.3389/fsoc.2024.1308029, 38505356 PMC10949981

[ref66] Kim U. Park Y. S. Park D. (2000). The challenge of cross-cultural psychology: the role of the indigenous psychologies. J. Cross-Cult. Psychol. 31, 63–75. doi: 10.1177/0022022100031001006

[ref67] Knibbe M. de Rijcke S. Penders B. (2025). Care for the soul of science: equity and virtue in reform and reformation. Cultures Sci. 8, 12–23. doi: 10.1177/20966083251329632

[ref68] Lakatos I. (1978). The Methodology of scientific Research Programmes (Philosophical Papers, Volume I). Cambridge University Press.

[ref69] Lamb D. Russell A. Morant N. Stevenson F. (2024). The challenges of open data sharing for qualitative researchers. J. Health Psychol. 29, 659–664. doi: 10.1177/13591053241237620, 38485917 PMC11141072

[ref70] Lash T. L. Vandenbroucke J. P. (2012). Commentary: should preregistration of epidemiologic study protocols become compulsory? Reflections and a counterproposal. Epidemiology 23, 184–188. doi: 10.1097/EDE.0b013e318245c05b, 22317802

[ref71] Lavelle J. S. (2023). Growth from uncertainty: understanding the replication 'crisis' in infant psychology. PhilSci Archive.

[ref72] Leonelli S. (2018). “Rethinking reproducibility as a criterion for research quality. Including a symposium on Mary Morgan: curiosity, imagination, and surprise,” in Research in the History of Economic Thought and Methodology, 36B, (Emerald Publishing), 129–146.

[ref73] Leonelli S. (2022). Open science and epistemic diversity: friends or foes? Philos. Sci. 89, 991–1001. doi: 10.1017/psa.2022.45

[ref74] Leung K. (2011). Presenting post hoc hypotheses as a priori: ethical and theoretical issues. Manag. Organ. Rev. 7, 471–479. doi: 10.1111/j.1740-8784.2011.00222.x

[ref75] Levy K. E. Johns D. M. (2016). When open data is a Trojan horse: the weaponization of transparency in science and governance. Big Data Soc. 3. doi: 10.1177/2053951715621568

[ref76] Lewandowsky S. Oberauer K. (2020). Low replicability can support robust and efficient science. Nat. Commun. 11:358. doi: 10.1038/s41467-019-14203-0, 31953411 PMC6969070

[ref77] Lilienfeld S. O. (2020). Embracing unpopular ideas: introduction to the special section on heterodox issues in psychology. Arch. Sci. Psychol. 8, 1–4. doi: 10.1037/arc0000072

[ref78] Linden A. H. Pollet T. V. Hönekopp J. (2024). Publication bias in psychology: a closer look at the correlation between sample size and effect size. PLoS One 19:e0297075. doi: 10.1371/journal.pone.0297075, 38359021 PMC10868788

[ref79] Linder C. Farahbakhsh S. (2020). Unfolding the black box of questionable research practices: where is the line between acceptable and unacceptable practices? Bus. Ethics Q. 30, 335–360. doi: 10.1017/beq.2019.52

[ref80] Lohse S. (2026). Reproducibility, questionable research practices and ethico-epistemic trade-offs in animal-based biomedicine. Eur. J. Philos. Sci. 16:25. doi: 10.1007/s13194-026-00727-y

[ref81] Longino H. E. (1990). Science as social Knowledge: Values and Objectivity in scientific Inquiry. Princeton University Press.

[ref82] MacEachern S. N. Van Zandt T. (2019). Preregistration of modeling exercises may not be useful. Comput. Brain Behav. 2, 179–182. doi: 10.1007/s42113-019-00038-x

[ref83] Malich L. Rehmann-Sutter C. (2022). Metascience is not enough: a plea for psychological humanities in the wake of the replication crisis. Rev. Gen. Psychol. 26, 261–273. doi: 10.1177/10892680221083876

[ref84] Mathur M. B. VanderWeele T. J. (2021). Estimating publication bias in meta-analyses of peer-reviewed studies: a meta-meta-analysis across disciplines and journal tiers. Res. Synth. Methods 12, 176–191. doi: 10.1002/jrsm.1464, 33108053 PMC7954980

[ref85] Metascience Can Improve Science (2025). Metascience can improve science — but it must be useful to society, too. Nature 643:304. doi: 10.1038/d41586-025-02065-0, 40629126

[ref86] Miller B. (2025). The Social Dimensions of scientific Knowledge: Consensus, Controversy, and Coproduction. Cambridge University Press.

[ref87] Miller J. D. Phillips N. L. Lynam D. R. (2025). Questionable research practices violate the American Psychological Association’s code of ethics. J. Psychopathol. Clin. Sci. 134, 113–114. doi: 10.1037/abn0000974, 39964511

[ref88] Mohseni A. (2020). HARKing: from misdiagnosis to misprescription. PhilSci Archive.

[ref89] Moody J. W. Keister L. A. Ramos M. C. (2022). Reproducibility in the social sciences. Annu. Rev. Sociol. 48, 65–85. doi: 10.1146/annurev-soc-090221-035954, 37284506 PMC10241466

[ref90] Moran C. Richard A. Wilson K. Twomey R. Coroiu A. (2022). I know it’s bad, but I have been pressured into it: questionable research practices among psychology students in Canada. Can. Psychol. 64, 12–24. doi: 10.1037/cap0000326, 27371692

[ref91] Morawski J. (2020). Psychologists’ psychologies of psychologists in a time of crisis. Hist. Psychol. 23, 176–198. doi: 10.1037/hop0000140, 31999140

[ref92] Morawski J. (2022). How to true psychology’s objects. Rev. Gen. Psychol. 26, 157–171. doi: 10.1177/10892680211046518

[ref93] Munafò M. R. Nosek B. A. Bishop D. V. Button K. S. Chambers C. D. du Percie Sert N. . (2017). A manifesto for reproducible science. Nat. Hum. Behav. 1:0021. doi: 10.1038/s41562-016-0021 33954258 PMC7610724

[ref94] Nagy T. Hergert J. Elsherif M. Wallrich L. Schmidt K. Waltzer T. . (2025). Bestiary of questionable research practices in psychology. Adv. Methods Pract. Psychol. Sci. 8. doi: 10.1177/25152459251348431

[ref95] Navarro D. (2020). Paths in strange spaces: a comment on preregistration. PsyArXiv. doi: 10.31234/osf.io/wxn58

[ref96] Nelson L. D. Simmons J. Simonsohn U. (2018). Psychology’s renaissance. Annu. Rev. Psychol. 69, 511–534. doi: 10.1146/annurev-psych-122216-011836, 29068778

[ref97] Nielsen M. Qiu K. (2018). A vision of metascience: an engine of improvement for the social processes of science. Science++ Project. Available online at: https://scienceplusplus.org/metascience/#learning-from-the-renaissance-in-social-psychology

[ref98] Nosek B. A. (2019). Strategy for culture change. Centre for Open Science. Available online at: https://www.cos.io/blog/strategy-for-culture-change

[ref99] Nosek B. A. (2025). Science becomes trustworthy by constantly questioning itself. PLoS Biol. 23:e3003334. doi: 10.1371/journal.pbio.3003334, 40749076 PMC12327609

[ref100] Nosek B. A. Lakens D. (2014). Registered reports. Soc. Psychol. 45, 137–141. doi: 10.1027/1864-9335/a000192

[ref101] Nosek B. A. Spies J. R. Motyl M. (2012). Scientific utopia: II. Restructuring incentives and practices to promote truth over publishability. Perspect. Psychol. Sci. 7, 615–631. doi: 10.1177/1745691612459058, 26168121 PMC10540222

[ref102] O’Boyle E. H. Banks G. C. Gonzalez-Mulé E. (2014). The chrysalis effect: how ugly initial results metamorphosize into beautiful articles. J. Manage. 43, 376–399. doi: 10.1177/0149206314527133

[ref103] O’Boyle E. H. Götz M. (2022). “Questionable research practices,” in Research Integrity: Best Practices for the social and Behavioral Sciences, eds. Jussim L. J. Krosnick J. A. Stevens S. T. (Oxford University Press), 260–294.

[ref104] O’Grady C. (2025). Science’s reform movement should have seen trump’s call for ‘gold standard science’ coming, critics say. Science adviser. Available online at: https://www.science.org/content/article/science-s-reform-movement-should-have-seen-trump-s-call-gold-standard-science-coming

[ref105] O’Rourke-Friel M. (2025). Social epistemology for individuals like us. Episteme 1–23, 1–23. doi: 10.1017/epi.2024.59, 41292463

[ref106] Oberauer K. Lewandowsky S. (2019). Addressing the theory crisis in psychology. Psychon. Bull. Rev. 26, 1596–1618. doi: 10.3758/s13423-019-01645-2, 31515732

[ref107] Open Science Collaboration (2015). Estimating the reproducibility of psychological science. Science 349:aac4716. doi: 10.1126/science.aac4716, 26315443

[ref108] Parker I. (2007). Critical psychology: what it is and what it is not. Soc. Personal. Psychol. Compass 1, 1–15. doi: 10.1111/j.1751-9004.2007.00008.x

[ref109] Pashler H. Harris C. R. (2012). Is the replicability crisis overblown? Three arguments examined. Perspect. Psychol. Sci. 7, 531–536. doi: 10.1177/1745691612463401, 26168109

[ref110] Pashler H. Wagenmakers E. J. (2012). Editors’ introduction to the special section on replicability in psychological science: a crisis of confidence? Perspect. Psychol. Sci. 7, 528–530. doi: 10.1177/1745691612465253, 26168108

[ref111] Paulus D. de Vries G. Janssen M. de Van Walle B. (2022). The influence of cognitive bias on crisis decision-making: experimental evidence on the comparison of bias effects between crisis decision-maker groups. Int. J. Disaster Risk Reduct. 82:103379. doi: 10.1016/j.ijdrr.2022.103379

[ref112] Penders B. (2024). Scandal in scientific reform: the breaking and remaking of science. J. Responsible Innov. 11. doi: 10.1080/23299460.2024.2371172

[ref113] Peterson D. Panofsky A. (2021a). Arguments against efficiency in science. Soc. Sci. Inf. 60, 350–355. doi: 10.1177/05390184211021383

[ref114] Peterson D. Panofsky A. (2021b). Self-correction in science: the diagnostic and integrative motives for replication. Soc. Stud. Sci. 51, 583–605. doi: 10.1177/03063127211005551, 33764246

[ref115] Peterson D. Panofsky A. (2023). Metascience as a scientific social movement. Minerva 61, 147–174. doi: 10.1007/s11024-023-09490-3

[ref116] Pethick S. Wass M. N. Michaelis M. (2025). Is there a reproducibility crisis? On the need for evidence-based approaches. Int. Stud. Philos. Sci. 38, 287–303. doi: 10.1080/02698595.2025.2538937

[ref117] Pham M. T. Oh T. T. (2021a). On not confusing the tree of trustworthy statistics with the greater forest of good science: a comment on Simmons et al.’s perspective on pre-registration. J. Consum. Psychol. 31, 181–185. doi: 10.1002/jcpy.1213

[ref118] Pham M. T. Oh T. T. (2021b). Preregistration is neither sufficient nor necessary for good science. J. Consum. Psychol. 31, 163–176. doi: 10.1002/jcpy.1209

[ref119] Pickett J. T. Roche S. P. (2018). Questionable, objectionable or criminal? Public opinion on data fraud and selective reporting in science. Sci. Eng. Ethics 24, 151–171. doi: 10.1007/s11948-017-9886-2, 28281156

[ref120] Popper K. R. (1976). “The logic of the social sciences,” in The Positivist Dispute in German Sociology, eds. Adorno T. W. Albert H. Dhrendorf R. Habermas J. Pilot H. Popper K. R. (Heineman), 87–104.

[ref121] Popper K. R. (1983). Realism and the aim of Science: From the Postscript to the logic of scientific discovery. Routledge.

[ref122] Popper K. R. (1994). “Knowledge and the body-mind problem,” in Defence of Interaction, (Routledge).

[ref7001] Popper K. R. (2002). The logic of scientific discovery. Routledge.

[ref123] Pownall M. (2024). Is replication possible for qualitative research? A response to Makel et al. (2022). Educ. Res. Eval. 29, 104–110. doi: 10.1080/13803611.2024.2314526

[ref124] Prosser A. M. B. Hamshaw R. Meyer J. Bagnall R. Blackwood L. Huysamen M. . (2023). When open data closes the door: Problematising a one size fits all approach to open data in journal submission guidelines. Br. J. Soc. Psychol. 62, 1635–1653. doi: 10.1111/bjso.12576, 36076340 PMC10946880

[ref125] Proulx T. Morey R. D. (2021). Beyond statistical ritual: theory in psychological science. Perspect. Psychol. Sci. 16, 671–681. doi: 10.1177/17456916211017098, 34240651

[ref126] Quintana D. S. Heathers J. A. J. (Hosts). (2023). 168: Meta-meta-science, Everything Hertz [Audio podcast]. doi: 10.17605/OSF.IO/CSJ3X

[ref127] Ramos M. L. F. (2025). Balanced examination of positive publication bias impact. Account. Res. 33. doi: 10.1080/08989621.2025.2538066, 40742139

[ref128] Ravn T. Sørensen M. P. (2021). Exploring the gray area: similarities and differences in questionable research practices (QRPs) across main areas of research. Sci. Eng. Ethics 27:40. doi: 10.1007/s11948-021-00310-z, 34136962

[ref129] Reinagel P. (2023). Is N-hacking ever OK? The consequences of collecting more data in pursuit of statistical significance. PLoS Biol. 21:e3002345. doi: 10.1371/journal.pbio.3002345 37910647 PMC10619921

[ref130] Reis D. Friese M. (2022). “The myriad forms of p-hacking,” in Avoiding Questionable Research Practices in Applied Psychology, eds. O'Donohue W. Masuda A. Lilienfeld S. (Spinger).

[ref131] Robinson-Greene R. (2017). The moral dimensions of the research reproducibility crisis. The Prindle Post. Available online at: https://digitalcommons.usu.edu/cgi/viewcontent.cgi?article=1615&context=lpsc_facpub

[ref132] Rooprai P. Islam N. Salameh J. P. Ebrahimzadeh S. Kazi A. Frank R. . (2023). Is there evidence of p-hacking in imaging research? Can. Assoc. Radiol. J. 74, 497–507. doi: 10.1177/08465371221139418, 36412994 PMC10338063

[ref133] Rubin M. (2017a). Do p values lose their meaning in exploratory analyses? It depends how you define the familywise error rate. Rev. Gen. Psychol. 21, 269–275. doi: 10.1037/gpr0000123

[ref134] Rubin M. (2017b). When does HARKing hurt? Identifying when different types of undisclosed post hoc hypothesizing harm scientific progress. Rev. Gen. Psychol. 21, 308–320. doi: 10.1037/gpr0000128

[ref135] Rubin M. (2020). Does preregistration improve the credibility of research findings? Quantitative Methods Psychol 16, 376–390. doi: 10.20982/tqmp.16.4.p376

[ref136] Rubin M. (2022). The costs of HARKing. Br. J. Philos. Sci. 73, 535–560. doi: 10.1093/bjps/axz050

[ref137] Rubin M. (2023a). Opening up open science to epistemic pluralism: comment on Bazzoli (2022) and some additional thoughts. Critical Metascience. doi: 10.31222/osf.io/dgzxa

[ref138] Rubin M. (2023b). Questionable metascience practices. J Trial Error 4, 5–20. doi: 10.36850/mr4, 42179678

[ref139] Rubin M. (2024). Type I error rates are not usually inflated. J Trial Error 4, 46–71. doi: 10.36850/4d35-44bd

[ref140] Rubin M. (2025a). The replication crisis is less of a “crisis” in Lakatos’ philosophy of science than it is in Popper’s. European journal for. Philos. Sci. 15. doi: 10.1007/s13194-024-00629-x, 30311153

[ref4001] Rubin M. (2025b). What is critical metascience and why is it important? Critical Metascience. doi: 10.59350/markrubin.167503816

[ref141] Rubin M. (2026a). A brief review of research that questions the impact of questionable research practices. PhilPapers. https://philpapers.org/rec/RUBABR

[ref142] Rubin M. (2026b). Critical metascience articles. Available online at: https://sites.google.com/site/markrubinsocialpsychresearch/replication-crisis/list-of-critical-metascience-articles

[ref143] Rubin M. (2026c). P-hacking inflates type I error rates in the error statistical approach but not in the formal inference approach. ArXiv. doi: 10.48550/arXiv.2602.21792

[ref144] Rubin M. Donkin C. (2024). Exploratory hypothesis tests can be more compelling than confirmatory hypothesis tests. Philos. Psychol. 37, 2019–2047. doi: 10.1080/09515089.2022.2113771

[ref145] Russell B. (1912). On the notion of cause. Proc. Aristot. Soc. 13, 1–26. http://www.jstor.org/stable/4543833

[ref146] Sacco D. F. Brown M. Bruton S. V. (2019). Grounds for ambiguity: justifiable bases for engaging in questionable research practices. Sci. Eng. Ethics 25, 1321–1337. doi: 10.1007/s11948-018-0065-x, 30259269

[ref147] Schimmack U. (2020). A meta-psychological perspective on the decade of replication failures in social psychology. Can. Psychol. 61, 364–376. doi: 10.1037/cap0000246

[ref148] Schooler J. (2014). Metascience could rescue the ‘replication crisis’. Nature 515:9. doi: 10.1038/515009a, 25373639

[ref149] Simmons J. P. Nelson L. D. Simonsohn U. (2011). False-positive psychology: undisclosed flexibility in data collection and analysis allows presenting anything as significant. Psychol. Sci. 22, 1359–1366. doi: 10.1177/0956797611417632, 22006061

[ref150] Simmons J. P. Nelson L. D. Simonsohn U. (2021). Pre-registration: why and how. J. Consum. Psychol. 31, 151–162. doi: 10.1002/jcpy.1208

[ref151] Simmons J. P. Nelson L. D. Simonsohn U. (2021a). Pre-registration is a game changer. But, like random assignment, it is neither necessary nor sufficient for credible science. J. Consum. Psychol. 31, 177–180. doi: 10.1002/jcpy.1207

[ref152] Stafford T. (2025). ECR metascientist happy hour. UK Reproducibility Network. Available online at: https://www.ukrn.org/2025/07/04/ecr-metascientist-happy-hour/

[ref153] Stanley T. D. Carter E. C. Doucouliagos H. (2018). What meta-analyses reveal about the replicability of psychological research. Psychol. Bull. 144, 1325–1346. doi: 10.1037/bul0000169, 30321017

[ref154] Stefan A. M. Schönbrodt F. D. (2023). Big little lies: a compendium and simulation of p-hacking strategies. R. Soc. Open Sci. 10:220346. doi: 10.1098/rsos.220346, 36778954 PMC9905987

[ref155] Steltenpohl C. N. Lustick H. Meyer M. S. Lee L. E. Stegenga S. M. Reyes L. S. . (2023). Rethinking transparency and rigor from a qualitative open science perspective. Journal of Trial & Error 4. doi: 10.36850/mr7

[ref156] Stroebe W. Strack F. (2014). The alleged crisis and the illusion of exact replication. Perspect. Psychol. Sci. 9, 59–71. doi: 10.1177/1745691613514450, 26173241

[ref157] Syed M. (2025). A Tale of Two Science Reform Movements: Get Syeducated Available online at: https://getsyeducated.substack.com/p/a-tale-of-two-science-reform-movements

[ref158] Szollosi A. Donkin C. (2021). Arrested theory development: the misguided distinction between exploratory and confirmatory research. Perspect. Psychol. Sci. 16, 717–724. doi: 10.1177/1745691620966796, 33593151

[ref159] Teymoori A. Trappes R. (2026). The recurrence of fundamental questions: a historical and philosophical analysis of major disciplinary crises in psychology. Rev. Gen. Psychol. 30, 195–211. doi: 10.1177/10892680261421875

[ref160] Tucker A. (2004). Our Knowledge of the past: A Philosophy of Historiography. Cambridge University Press.

[ref161] Turner M. E. Pratkanis A. R. (1998). A social identity maintenance model of groupthink. Organ. Behav. Hum. Decis. Process. 73, 210–235. doi: 10.1006/obhd.1998.2757, 9705803

[ref162] UK Metascience Unit. (2025). A year in metascience: the past, present and future of UK metascience (annex). Available online at: https://assets.publishing.service.gov.uk/media/685bcd40c07c71e5a87097d1/the-past-present-future-of-uk-metascience.pdf

[ref163] Ulpts S. (2024). Responsible assessment of what research? Beware of epistemic diversity! Meta-Psychology 8. doi: 10.15626/MP.2023.3797

[ref164] Ulpts S. Bartscherer S. (Convenors). (2025). Critical metascience: Does metascience need to change? Metascience 2025 Preconference Virtual Symposium. Available online at: https://cassyni.com/events/ADvXMC3V9NYJguU8AEKHGR

[ref165] Ulpts S. Bartscherer S. F. Field S. M. Penders B. (2025). The social replication of replication: moving replication through epistemic communities. SocArXiv. doi: 10.31235/osf.io/pqc4v_v1

[ref166] Ulrich R. Miller J. (2020). Questionable research practices may have little effect on replicability. eLife 9:e58237. doi: 10.7554/eLife.58237, 32930092 PMC7561355

[ref167] Uygun Tunç D. Tunç M. N. Eper Z. B. (2023). Is open science neoliberal? Perspect. Psychol. Sci. 18, 1047–1061. doi: 10.1177/17456916221114835, 36476075 PMC10475209

[ref168] Van Aert R. C. Wicherts J. M. Van Assen M. A. (2019). Publication bias examined in meta-analyses from psychology and medicine: a meta-meta-analysis. PLoS One 14:e0215052. doi: 10.1371/journal.pone.0215052, 30978228 PMC6461282

[ref169] van Rooij I. Baggio G. (2021). Theory before the test: how to build high-verisimilitude explanatory theories in psychological science. Perspect. Psychol. Sci. 16, 682–697. doi: 10.1177/1745691620970604, 33404356 PMC8273840

[ref170] Vancouver J. N. (2018). In defense of HARKing. Ind. Organ. Psychol. 11, 73–80. doi: 10.1017/iop.2017.89

[ref171] Vazire S. Holcombe A. O. (2022). Where are the self-correcting mechanisms in science? Rev. Gen. Psychol. 26, 212–223. doi: 10.1177/10892680211033912

[ref172] Vazire S. Nosek B. (2023). Introduction to special topic “is psychology self-correcting? Reflections on the credibility revolution in social and personality psychology”. Soc. Psychol. Bull. 18, 1–4. doi: 10.32872/spb.12927

[ref173] Wagenmakers E. J. Wetzels R. Borsboom D. van der Maas H. L. Kievit R. A. (2012). An agenda for purely confirmatory research. Perspect. Psychol. Sci. 7, 632–638. doi: 10.1177/1745691612463078, 26168122

[ref174] Walkup J. (2021). Replication and reform: vagaries of a social movement. J. Theor. Philos. Psychol. 41, 131–133. doi: 10.1037/teo0000171

[ref175] Wegener D. T. Pek J. Fabrigar L. R. (2024). Accumulating evidence across studies: consistent methods protect against false findings produced by p-hacking. PLoS One 19:e0307999. doi: 10.1371/journal.pone.0307999, 39208346 PMC11361653

[ref176] Whitaker K. Guest O. (2020). # bropenscience is broken science. Psychologist 34, 33–37. doi: 10.1080/02690055.2019.1577602, 37339054

[ref177] Wicherts J. M. Veldkamp C. L. Augusteijn H. E. Bakker M. Van Aert R. C. Van Assen M. A. (2016). Degrees of freedom in planning, running, analyzing, and reporting psychological studies: a checklist to avoid p-hacking. Front. Psychol. 7:1832. doi: 10.3389/fpsyg.2016.01832, 27933012 PMC5122713

[ref178] Wingen T. Berkessel J. B. Englich B. (2020). No replication, no trust? How low replicability influences trust in psychology. Soc. Psychol. Personal. Sci. 11, 454–463. doi: 10.1177/1948550619877412

